# Toxic, Radical Scavenging, and Antifungal Activity of *Rhododendron tomentosum* H. Essential Oils

**DOI:** 10.3390/molecules25071676

**Published:** 2020-04-05

**Authors:** Asta Judzentiene, Jurga Budiene, Jurgita Svediene, Rasa Garjonyte

**Affiliations:** 1 State Research Institute Center for Physical Sciences and Technology, Department of Organic Chemistry; Sauletekio Ave. 3, LT–10222 Vilnius, Lithuania; jurga.budiene@ftmc.lt (J.B.); rasa.garjonyte@ftmc.lt (R.G.); 2 Nature Research Centre, Laboratory of Biodeterioration Research; Akademijos 2, LT–08412 Vilnius, Lithuania; jurgita.svediene@gamtc.lt

**Keywords:** *Rhododendron tomentosum* H., *Ericaceae*, essential oil composition, antifungal activity, antioxidant tests, toxicity in vivo, amperometry

## Abstract

The chemical composition of eight (seven shoot and one inflorescence) essential oils (EOs) of *Rh. tomentosum* H. plants growing in Eastern Lithuania is reported. The plant material was collected during different phases of vegetation (from April to October). The oils were obtained by hydrodistillation from air-dried aerial parts (leaves and inflorescences). In total, up to 70 compounds were identified by GC−MS and GC (flame-ionization detector, FID); they comprised 91.0 ± 4.7%–96.2 ± 3.1% of the oil content. Sesquiterpene hydrocarbons (54.1 ± 1.5%–76.1 ± 4.5%) were found to be the main fraction. The major compounds were palustrol (24.6 ± 2.6%–33.5 ± 4.4%) and ledol (18.0 ± 2.9%–29.0 ± 5.0%). Ascaridol isomers (7.0 ± 2.4%–14.0 ± 2.4% in three oils), myrcene (7.2 ± 0.3% and 10.1 ± 1.3%), lepalol (3.3 ± 0.3% and 7.9 ± 3.0%), and cyclocolorenone isomers (4.1 ± 2.5%) were determined as the third main constituents. The toxic activity of marsh rosemary inflorescence and shoot oils samples was evaluated using a brine shrimp (*Artemia* sp.) bioassay. LC_50_ average values (11.23–20.50 µg/mL) obtained after 24 h of exposure revealed that the oils were notably toxic. The oil obtained from shoots gathered in September during the seed-ripening stage and containing appreciable amounts of palustrol (26.0 ± 2.5%), ledol (21.5 ± 4.0%), and ascaridol (7.0 ± 2.4%) showed the highest toxic activity. Radical scavenging activity of *Rh. tomentosum* EOs depended on the plant vegetation stage. The highest activities were obtained for EOs isolated from young shoots collected in June (48.19 ± 0.1 and 19.89 ± 0.3 mmol/L TROLOX (6-hydroxy-2,5,7,8-tetra-methylchromane-2-carboxylic acid) equivalent obtained by, respectively, ABTS^•^^+^ (2,2′-amino-bis(ethylbenzothiazoline-6-sulfonic acid) diammonium salt) and DPPH^•^(2,2-diphenyl-1-picrylhydrazyl) assays). Agar disc diffusion assay against pathogenic yeast *Candida parapsilosis* revealed the potential antifungal activity of EOs. An alternative investigation of antifungal activity employed mediated amperometry at yeast *Saccharomyces cerevisiae*-modified electrodes. The subjection of yeast cells to vapors of EO resulted in a three to four-fold increase of electrode responses due to the disruption of yeast cell membranes.

## 1. Introduction

*Rhododendron tomentosum* Harmaja (previously entitled by several different names [1], most frequently *Ledum palustre* Linnaeus), family *Ericaceae,* commonly is called marsh rosemary, marsh tea, or marsh Labrador tea. It is a fragrant woody evergreen small shrub (up to 1 m in height), distributed in northern and central Europe, northern parts of Asia, and North America. *Rh. tomentosum* H. is a perennial plant, growing in a peaty soil and forming colonies. The plant known under the name Devil’s Broom growth throughout all the territory of Lithuania: in marshes, coniferous forests, peat bogs, and in the sandy pine forests on the Baltic seashore.

*Rh. tomentosum* has been used in folk medicine or homeopathy due to the diuretic, diaphoretic, emetic, abortifacient, anti-inflammation, narcotic, pectoral, tonic etc., properties for healing infections, female disorders, arthrosis, rheumatism, bronchitis, insect bites, cold, cough, dysentery, gout, tuberculosis, different pains, skin problems, diabetes symptoms, and other illnesses both externally and internally [1]. On the basis of the scientific research, diverse bioactivities, such as antimicrobial, antioxidant, anticancer, antidiabetic, and antioxidant have been established [2,3]. Volatile components of *Rh. tomentosum* have been found to possess repellent properties effective against bedbugs and clothing moths [4].

*Rh. tomentosum* plants synthesize a high content of terpenoids in glandular trichomes and emit a strong characteristic smell, which attracts bees and other pollinating insects. The smell may cause headaches to some people. The chemical compositions of the essential oils (EOs) of the entire plant or separated aerial parts (mostly shoots) of *Rh. tomentosum* have been studied in some European countries [5,6,7,8,9,10,11,12,13,14,15,16,17,18,19], the Far East and Siberia [20,21,22,23,24,25], and Asian countries [17,26,27,28,29,30,31,32,33,34,35,36,37]. A lot research works have been focused on the evaluation of bioactivities of marsh rosemary extracts. It was shown that *Rh. tomentosum* extracts, essential oils, or emitted volatiles influence herbivores’ behavior [9,10,38,39,40] and the growth of neighboring plants [24], have an impact on polypropylene plastic degradation [31], and possess analgesic [41], antibacterial [42], anticancer [43,44], antidiabetic [45,46], antifungal [47,48], anti-inflammatory [41,42,49,50,51], antioxidant [33,46], antimicrobial [33,52], anti-proliferative, and pro-apoptotic [19], antithrombin [43], hepatoprotective [53], toxic [54], and radioprotective [55,56] properties (see Supplementary materials: Table S1 for details).

The aim of this work is to relate the chemical composition of Lithuanian marsh rosemary (*Rh. tomentosum* H. at various vegetative stages) essential oils to their toxic (*Artemia sera* larvae lethality test), radical scavenging ABTS^•^^+^ (2,2′-amino-bis(ethylbenzothiazoline-6-sulfonic acid) diammonium salt), DPPH^•^ (2,2-diphenyl-1-picrylhydrazyl), and TROLOX (6-hydroxy-2,5,7,8-tetra-methylchromane-2-carboxylic acid) equivalent assay) and antifungal (against pathogenic *Candida parapsilosis* and model organism unicellular fungus *Saccharomyces cerevisiae*) activities.

Although the number of publications concerning various bioactivities of *Rh. tomentosum* extracts is large, the toxicity tests of wild marsh rosemary EOs, using brine shrimp larvae assay, and antifungal tests of the oils employing mediated amperometry have not been performed previously.

## 2. Results

### 2.1. Chemical Composition of Essential Oils

The main chemical compositions (including constituents ≥3.0%) of seven shoot (Sh) and one inflorescence (Fl) essential oils of *Rh. tomentosum* H. growing in Samanis forest marshes (Eastern part of Lithuania) are given in Table 1.

The plant material was collected from April (at the shoot-growing stage) to October (seed full-ripening phase). Palustrol (24.6 ± 2.6–33.5 ± 4.4% in all oil samples), ledol (18.0 ± 2.9–29.0 ± 5.0% in all samples), ascaridol isomers (7.0 ± 2.4–14.0 ± 2.4% in three oils), myrcene (7.2 ± 0.3 and 10.1 ± 1.3%), lepalol (3.3 ± 0.3 and 7.9 ± 3.0%), and cyclocolorenone isomers (4.1 ± 2.5%) were determined among the three major constituents in the oils. Palustrol and ledol was the first or second dominant compound in all the oils, and the total average quantity of these two sesquiterpene alcohols comprised on average 42.6–62.5% of all the oil content. The third position was attributed to the rest of the compounds listed above. *iso*-Ascaridol (most probably the sum of ascaridol and *iso*-ascaridol or even ascaridol alone) was the third main constituent in shoot oils obtained from marsh rosemary in July, August, and September (5–7 Sh), while an average content of this compound varied from 0.1% to 5.3% in the rest of the samples. Monoterpene hydrocarbon myrcene occupied the third position in inflorescence (3 Fl May) and shoot (4 Sh June) oils (10.1 ± 1.3 and 7.2 ± 0.3%, respectively). One shoot oil (8 Sh) collected during the seeding stage in October was characterized by an appreciable amount (7.9 ± 0.3%) of heteroaromatic compound lepalol, while an average quantity of this constituent varied from 0.1% to 3.3% in the rest of the samples. Two cyclocolorenone isomers (4.1 ± 2.5%) occupied the third position in the shoot oil (2 Sh) of plants gathered during the flowering stage (Table 1).

### 2.2. Toxic Activity

A toxicity test employing brine shrimp (*Artemia sera*) larvae and eight EOs (seven shoot and one inflorescence oil) revealed that all the *Rh. tomentosum* EOs under study were notably toxic. LC_50_ and LC_95_ values were in the range 11.23–20.50 and 34.00–76.07 µg/mL, respectively (Table 2). 

Essential oils obtained from shoots gathered in September (7 Sh seed-ripening stage) that contained appreciable amounts of palustrol (26.0 ± 2.5%), ledol (21.5 ± 4.0%), and ascaridol (7.0 ± 2.4%) appeared to be the most toxic (Table 2). In contrary, inflorescence oil (3 Fl, Table 2) was comprised of almost the same quantities of palustrol (30.0 ± 1.6%) and ledol (23.3 ± 2.3%), but 10.1 ± 1.3% of myrcene and a minor amount (1.4 ± 0.6%) of ascaridol isomers was evaluated as less toxic (Table 2). 

### 2.3. Radical Scavenging Activity

The ABTS^•^^+^ and DPPH^•^ assays are commonly employed to evaluate the ability of antioxidants to scavenge free radicals. The antioxidant activities of tested four Rh. tomentosum shoot EOs assayed by ABTS^•^^+^ varied depending on the plant vegetation stage (Table 3).

The highest antioxidant activity (48.19 ± 0.1 mmol/L TROLOX) was obtained using young shoot (sample 4 Sh collected in June) essential oil. The older the plant, the lower ability of oil to scavenge free radicals (30.35 ± 0.03 and 16.25 ± 0.2 mmol/L for shoot oils 7 Sh and 8 Sh of plants collected in autumn) was determined.

The antioxidant activities of six wild marsh rosemary shoot EOs and one flower EO tested by DPPH^•^ assay also varied depending on the plant vegetation stage (Table 4).

The lowest values (5.86 ± 0.07 mmol/L TROLOX) were obtained for flower oil (3 Fl), while summer shoot oils possessed the highest activities (19.89 ± 0.3 and 17.82 ± 0.5 mmol/L TROLOX for 4 Sh and 5 Sh, respectively).

### 2.4. Antifungal Activity

#### 2.4.1. Antifungal Activity of EO against Yeast Tested by Agar Disc Diffusion Method

The antifungal activity of essential oils against microorganisms is often assayed by the agar disc diffusion method [58]. The diameters of the inhibition zones formed on agar layers with inoculated tested cultures reflect the capabilities of EOs to affect their growth: the larger the inhibition zone that is obtained, the more effective the antimicrobial activity that is ascribed to the EO. Agar plates were inoculated with Candida parapsilosis. Then, the filter paper discs (6 mm in diameter) impregnated with 10 µL of diluted EO (8 Sh) and nystatin (an agent used to treat Candida infections) as a positive control were placed on the agar surfaces.

**Figure 1:**
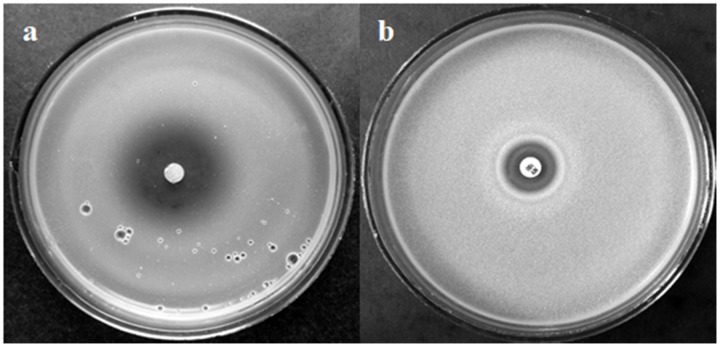
Inhibition of *C. parapsilosis* growth by *Rh. tomentosum* essential oil (EO) (8 Sh) (**a**) and nystatin (**b**).

After 48 h at 30 °C, the inhibition zone around an EO-impregnated paper disc was about 30 mm (Figure 1a), i.e., three-fold larger compared to that around a commercial nystatin-impregnated paper disc (Figure 1b). Besides, the density of the *Candida parapsilosis* lawn in a plate containing EO was lower than in the plate with nystatin, indicating the suppressing effect of EO vapors on *C. parapsilosis* growth. 

The attempts to use this agar disc diffusion assay to evaluate the antifungal activity of *Rh. tomentosum* against *Saccharomyces cerevisiae* were unsuccessful. The growth was completely suppressed at high EO concentrations, whereas the results were not reproducible when using diluted samples.

#### 2.4.2. Effect of *Rh. Tomentosum* EO Volatile Compounds on Yeast *S. cerevisiae* Membrane Permeability

**Figure 2:**
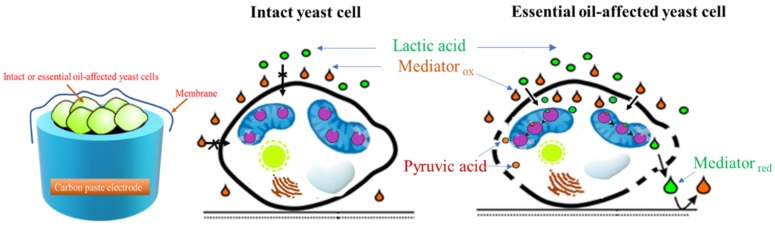
Scheme of amperometric measurements.

Instead of agar disc assay, mediated amperometry was used as an alternative. The effect of the essential oil on the yeast membrane permeability was monitored by measuring potassium ferricyanide-mediated current responses to lactic acid at yeast-modified electrodes treated with vapors of EO (a schematic image of amperometric measurements is presented in Figure 2). 

**Figure 3:**
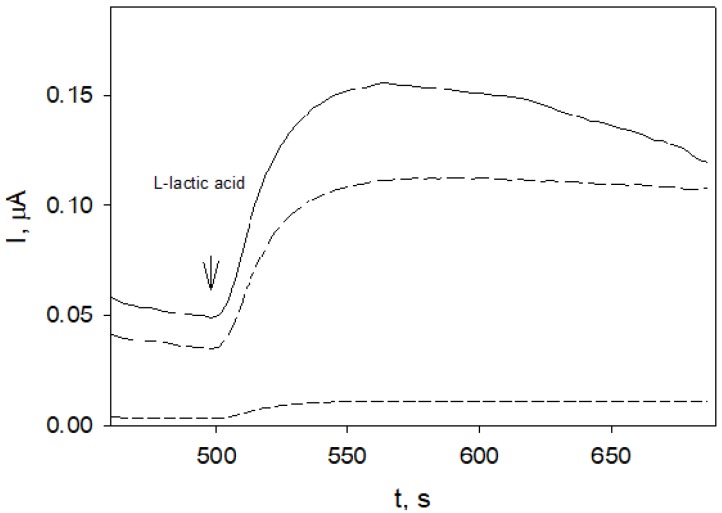
Amperometric responses of yeast-modified electrodes to 0.2 mmol/L *L*-lactic acid: not affected by vapors (short-dashed line), after 20 min (long-dashed line) and after 40 min (solid line) of subjection to vapors of *Rh. tomentosum* EO (8 Sh); phosphate buffer pH 7.3 contained 0.5 mmol/L K_3_[Fe(CN)_6_]; operating potential 0.3 V.

Obviously higher current responses at affected electrodes (Figure 3, solid and medium-dashed lines) compared to that obtained at a non-affected electrode (Figure 3, short-dashed line) indicated the increase in membrane permeability to lactic acid and/or mediator K_3_[Fe(CN)_6_]. 

**Figure 4:**
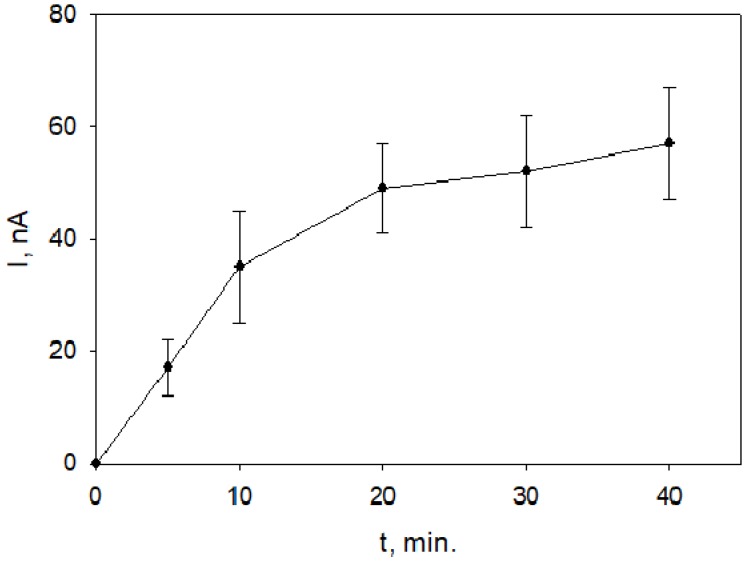
Dependence of yeast-modified electrode responses to 0.2 mmol/L L-lactic acid on the time of electrode subjection to vapors of *Rh. tomentosum* EO (8 Sh), phosphate buffer pH 7.3 contained 0.5 mmol/L K_3_[Fe(CN)_6_]; operating potential 0.3 V.

The dependence of maximal electrode responses to 0.2 mmol/L *L*-lactic acid on the time of electrode subjection to vapors of *Rh. tomentosum* EO is given in Figure 4.

## 3. Discussion

In order to obtain sufficient amounts of wild rosemary EOs, a hydrodistillation time of 2 h was chosen. Previously, it was proven that the extension of distillation time from 2 to 4 h has increased *Rh. tomentosum* oil yield without significant changes in the composition of the oils [12].

The yields of *Rh. tomentosum* EOs varied significantly (almost three-fold) depending on the plant vegetation stage. During plant growing, the yields were the lowest 0.59% (*v*/*w*) in April, and increased to maximum values of 1.73% and 1.76% (*v*/*w*) in June and July (at the end of flowering), respectively. Thereafter, the yields decreased to 1.36% (*v*/*w*) in October, at full seeding stage. The differences in the content of EOs could be explained by temperature and sun illumination intensity as well.

Totally, up to 70 constituents were identified in the *Rh. tomentosum* EOs; they comprised from 91.0 ± 4.7% to 96.2 ± 3.1% of total oil content. Oxygenated sesquiterpene hydrocarbons (54.1 ± 1.5%–76.1 ± 4.5%) were found to be the main fraction. A detailed chemical composition of the EOs of marsh rosemary (collected in 2007) from Samanis forest marsh was presented in the previous work [13].

All the oils were characterized by major compounds of aromadendrane skeleton, ledol, and palustrol. The largest quantities of these two principal compounds were determined in the beginning (in April and May) and at the end of vegetation (October), while the lowest amount of them was detected in June, at the beginning of seed formation (Table 1). In our study, quantitative changes of ledol and palustrol during various vegetative phases were not very remarkable, whereas concentrations of these compounds differed several times in the EOs of *Rh. tomentosum* plants collected in June and October in Poland [18]. It should be mentioned that the ledol + palustrol chemotype is common for the plants of European and Asian origin.

It is known that ascaridol is a heat-sensitive compound, which undergoes thermal isomerization and can be converted fully or partially to *iso*-ascaridole during GC analysis. *iso*-Ascaridol (most probably the sum of ascaridol and *iso*-ascaridol or even ascaridol alone) was determined as the third main constituent in shoot oils obtained from marsh rosemary in July, August, and September (5–7 Sh), during pre-seed and seed-ripening stages. The content of *iso*-ascaridol changed drastically (from 0.1 ± 0.1% to 14.0 ± 2.4%) during different vegetative stages. Although only *iso*-ascaridol was determined chromatographically, it is quite possible that actually both isomers *iso*-ascaridol and ascaridol or ascaridol alone exist in the tested oils. Formerly, in order to clarify this phenomenon, a quantitative determination of ascaridol isomers in the *Rh. tomentosum* shoot essential oil was performed by ^13^C NMR spectroscopy [15]. Spectra were recorded at room temperature, and under these conditions, neither degradation nor thermal isomerization occurred. Higher ascaridol concentration (4.0%) was determined by ^13^C NMR compared to those (0.6%) determined chromatographically by GC (flame-ionization detector, FID) [15]. Ascaridol also has been identified as a predominant compound in the marsh rosemary plants growing in some countries, such as Finland, Sweden, and China [6,10,26]. 

Two oils (3 Fl, May) and (4 Sh, June) contained myrcene as the third major constituent (Table 1). This monoterpene hydrocarbon has been determined frequently among the main constituents in marsh rosemary plants investigated in other countries [6,9,11,12,22,30]. 

Furyl-containing compounds such as lepaline, lepalone, lepalol, and 2-methyl-5-(3-furyl)-3-penten-2-ol were present in all the studied oils (Table 1). The quantities of these furyl monoterpenoids increased in the autumn months. An appreciable amount (7.9 ± 0.3%) of lepalol was determined in shoot oil (8 Sh) obtained from marsh rosemary gathered in October. Lepalol in a quantity of 3.3 ± 0.3% was found as the third major constituent in the shoot oil (1 Sh) in April. 

Cyclocolorenone + *epi*-cyclocolorenone (4.1 ± 2.5%) occupied the third position in the shoot oil (2 Sh) of plants gathered during the flowering stage (Table 1). Cyclocolorenone derivatives were determined as the dominating constituents in marsh rosemary plants of Russian origin (Leningrad region) [8].

To the best to our knowledge, shyobunone isomers are quite rare for *Rh. tomentosum* EOs. This sesquiterpenoid was identified in shoots during June, July, and August among minor constituents (up to 0.5%) (Table 1). Recently, shyobunones have been determined in appreciable amounts (up to 5.7%) in the oils of marsh rosemary of Polish origin [18].

The metabolic pathway of some selected aromadendranes, which are the main components of *R. tomentosum* EOs, has been reported [59]. It was proposed that the biosynthesis of ledol takes place directly from *allo*-aromadendrene or through *allo*-aromadendrene epoxide. Possibly, the *allo*-aromadendrene is also a precursor of palustrol; the pathway proceeds through ledene oxide (II) [17]. On other hand, it was suggested that (+)-bicyclogermacrene could be a potential platform intermediate for several aromadendrane sesquiterpenoids, such as ledene, viridiflorol, palustrol, and spathulenol [60]. Obviously, there could be several possible biosynthesis pathways of ledol and palustrol in the plants. On the basis of the minor amounts of *allo*-aromadendrene determined in our research and the absence of bicyclogermacrene, we can hypothesize that the ledol and palustrol biosynthetic pathway arises from *allo*-aromadendrene. α-Terpinene is a precursor of ascaridol [61]; the synthesis of the latter compound takes place in presence of oxygen and light. It is known that the laboratory synthesis of monoterpenoid ascaridol is performed from α-terpinene as well.

Toxicity tests *in vivo* performed using brine shrimp (*Artemia sera)* larvae revealed that LC_50_ average values varied from 11.23 to 20.50 µg/mL. Marsh rosemary inflorescence oil (3 Fl) appeared to be less toxic (LC_50_ = 20.50 µg/mL), while the essential oil obtained from shoots gathered in September (7 Sh, seed ripening stage) and containing appreciable amounts of palustrol (26.0 ± 2.5%), ledol (21.5 ± 4.0%), and ascaridol (7.0 ± 2.4%) was the most toxic (LC_50_ = 11.23 µg/mL) (Table 1 and Table 2). According to the available literature data, research studies devoted to the *Rh. tomentosum* toxicity *in vivo* are very scarce (Table S1). Marsh rosemary shoot extracts in different organic solvents were tested on mice by intraperitoneal and intragastric administration [54]. Palustrol obtained from the soft coral *Sarcophyton troheliophorum* has exhibited high toxic activity (LD_50_ ≥ 11.1 µM) in brine shrimp assays (against *Artemia salina*) [62]. *Hagenia abyssinica* flower oil containing 58.57% of ledol exhibited trypanocidal (against *Trypanosoma brucei*) activities with IC_50_ values of 42.30 μg/mL [63]. Inhibitory larvicidal efficacy (LC_50_) of essential oils from *Salvia dorisiana*, including appreciable amount (45.8%) of ledol against fourth instar larvae of *Aedes albopictus* Skuse was found to be 76.7 ppm [64].

The abilities of plant extracts or separate compounds to scavenge ABTS cation radicals and DPPH radicals are frequently used to evaluate antioxidant activity. Young shoot essential oil (4 Sh, June) were the most active (48.19 ± 0.1 mmol/L TROLOX sample). The older the plant, the lower the ability of its second metabolite to scavenge ABTS^•^^+^, i.e., 30.35 ± 0.03 and 16.25 ± 0.2 mmol/L for shoot oils (**7** Sh and 8 Sh) of plants collected in September and October, respectively. A similar tendency was observed in DPPH^•^ assay. Values of the TROLOX equivalent ranged from 5.86 ± 0.07 in flower oils to 19.89 ± 0.3 in shoot oils collected in June. Young shoot essential oil (4 Sh, June) were the most active in DPPH^•^ assay as well. 

Due to different methods used for the evaluation of antioxidant activity of *Rh. tomentosum* extracts [33,45], it is complicated to compare our results with already published data. Marsh rosemary EO, containing major compounds sabinene, terpinen-4-ol, and myrtenal, strongly reduced DPPH^•^ formation and exhibited a hydroxyl radical scavenging effect in the deoxyribose system, and it inhibited the nonenzymatic lipid peroxidation of rat liver homogenate [33]. It was revealed that *Rh. tomentosum* extract containing an appreciable content of polyphenols possessed strong antioxidant activity, which was close to that of ascorbic acid [45].

The antifungal activity of *Rh. tomentosum* essential oil (8 Sh, one of the most toxic) was tested against *C. parapsilosis.* Infections caused by *Candida albicans* and non-*albicans Candida* species remain one of the most common types of fungal infections. A previous investigation of the antifungal/antimicrobial activities of *Rh. tomentosum* EOs [33,45] revealed varying (from mild to strong) effects against *C*. *albicans*. *C. parapsilosis* is of particular importance, as it is able to form tenacious biofilms on central venous catheters and other implanted devices, thus threatening patients who have undergone medical interventions [65]. The agar disc diffusion method revealed the large effect of tested EOs on the formation of transparent inhibition zones and lowering the density of *C. parapsilosis* lawn (Figure 1). This pilot investigation with *Candida* species suggests that the EOs of *Rh. tomentosum* possess potential antifungal activity.

*S. cerevisiae*, the unicellular fungus, is frequently employed as a model organism in scientific research to investigate the mechanisms of various physiological functions within eukaryotic cells and to understand their interactions with the surrounding environment. *S. cerevisiae* usually are considered non-pathogenic; nevertheless, some information related to *S. cerevisiae* as one of the emerging pathogens has been published [66,67]. Since the agar disc assay experiments with *S. cerevisiae* were not successful, mediated amperometry was used as an alternative.

Among several mechanisms responsible for the antifungal activity of EOs [68], the disruption of cell membranes is frequently determined. The yeast cell envelope consists of a plasma membrane and cell wall [69]. A relatively thick cell wall composed mostly from polysaccharides, chitin, and proteins is responsible for the cell’s resistance to mechanical stress and presents no real barrier to the diffusion to small molecules and ions. The plasma membrane forms a relatively impermeable barrier for hydrophilic molecules. Membrane permeability can be increased by cell treatment with solvents, detergents, salts, cell freezing and thawing [70], or by electropermeabilization [71]. Recently, the effect of essential oil of *Mentha piperita* L. on various yeasts through the perturbation of different physiological functions (including membrane permeability) was reported [72]. As an alternative to the sophisticated flow cytometry presented in the latter publication, we have used a mediated amperometry to evaluate the permeability of yeast membrane. An amperometric approach to investigate the permeability of yeast membrane is based on the *S. cerevisiae*-modified electrode responses to L-lactic acid [73] according to the scheme:

*L*-lactic acid + flavocytochrome b_2(ox)_ →pyruvic acid + flavocytochrome b_2(red)_ (1)

flavocytochrome b_2(red)_ + mediator_(ox)_→flavocytochrome b_2(ox)_ + mediator_(red)_ (2)

mediator_(red)_→mediator_(ox)_ + e^−^ (3)

where Equations (1) and (2) are chemical reactions of flavocytochrome b_2_ with the lactic acid and mediator, respectively, and Equation (3) is the electrochemical oxidation of the reduced form of mediator (schematic image is presented in Figure 2).

The function of mediator is to shuttle the electrons between the electrode and the redox center of the enzyme flavocytochrome b_2_ located in the inter-membrane space of yeast mitochondria. Potassium ferricyanide appeared to be very suitable for this purpose [73].

Without EO treatment, the current responses to 0.2 mmol/L *L*-lactic acid at the yeast-modified electrode were obviously lower (Figure 3, short-dashed line) compared to those obtained when yeast-modified electrodes were subjected to vapors of EO (Figure 3, long-dashed and solid lines). The decrease of the current response during measurement (Figure 3, solid line) possibly could indicate that a longer time (40 min) of subjection to vapors has affected the enzyme as well. The effect of *Rh. tomentosum* EO vapors on yeast was observed already in 10 min and increased with time (Figure 4). A maximal effect was observed after about 20 min and practically did not change during the tested time (up to 40 min). This was a relatively short time needed to observe the effect of volatile compounds on yeast membrane permeability. For comparison, yeast-modified electrode subjection to the vapors of *Juniperus communis* L. EOs for up to 40 min practically had no effect on electrode responses to lactic acid. The fast effect of vapors of *Rh. tomentosum* essential oil on yeasts could be the possible reason why inhibition tests on agar were not successful. The research of the effects of various EOs on yeast membrane permeability is in progress. Dynamics of yeast-electrode responses to lactic acid could serve as a measure of EO antifungal activity. The shortcoming of the proposed amperometric method is that it can be applied for unicellular yeasts possessing relatively easily inducible changes in membrane permeability. 

## 4. Materials and Methods

### 4.1. Plant Material

*Rhododendron tomentosum* plant shoots and inflorescences (up to 1.0 kg) were collected at various vegetative stages (from April to October, during several years) in the forest Samanis (Utena district, Eastern Lithuania) marshes. The habitat of investigated population is depicted on the geographic information system map (Figure S1). A voucher specimen has been deposited in the Herbarium of the Institute of Botany (BILAS), number 68889. Plant material was dried at room temperature (20–25 °C); leaves and inflorescences were separated before drying. 

### 4.2. Essential Oil Preparation 

The essential oils were extracted from air-dried material (50g each) by hydrodistillation for 2 h in a Clevenger-type apparatus according to the *European Pharmacopoeia*. The ratio of plant material to water was 1:20. A yellow-gray, greasy mass with a sweet characteristic odor was obtained. The yields of the oils ranged from 0.59% to 1.76% (*v*/*w*, on a dry weight basis). The obtained oils were dried over anhydrous sodium sulfate, kept in closed dark vials and stored in a refrigerator at −18 °C. In order to obtain appropriate chromatograms, the pure oil samples were diluted dozens of times with a mixture of pentane and diethyl ether (1:1) just before analyses. 

### 4.3. GC (Flame-Ionization Detector FID) Analysis

Quantitative analyses of the essential oils were carried out by GC on DB-5 ((5%-phenyl)-methylpolisiloxane; 50 m × 0.32 mm × 0.25 μm) and HP-FFAP (polyethylene glycol 30 m × 0.25 mm i.d., film thickness 0.25 μm) capillary columns using an HP 5890II chromatograph equipped with a FID. The GC oven temperature was programmed as follows: from 60 °C (isothermal for 3 min) increased to 160 °C (isothermal for 2 min) at a rate of 5 °C/min, then increased to 250 °C at a rate of 10 °C/min, and the final temperature was kept for 3 min. The temperature of the injector and detector was maintained at 250 °C. The flow rate of carrier gas (hydrogen) was 1 mL/min. At least two repetitions (*n* ≥ 2) per analysis were performed. Injection volume was 2 µL. Retention indices were determined relative to the retention times of a series of n-alkanes. The relative proportions of the oil constituents were expressed as percentages obtained by peak area normalization, all relative response factors being taken as one.

### 4.4. GC-MS Analysis

Analyses were carried out using an HP 5890 gas chromatograph equipped with an HP 5971 mass selective detector and an HP 7673 split/splitless (splitless 0.75 min) injector on the same capillary column DB-5. The conditions of chromatographic separation were the same as for GC (FID) analysis. The temperature of the injector and detector was 250 °C. The flow rate of carrier gas (helium) was 1 mL/min. At least two repetitions (*n* ≥ 2) per analysis were performed. Injection volume 2 µL. Mass spectra in electron mode were generated at 70 eV, 0.97 scans/second, mass range 35–650 m/z.

### 4.5. Identification of Individual Components

The percentage composition of the essential oils was computed from GC peak areas without correction factors. Qualitative analysis was based on comparison of retention indexes on both columns, co-injection of some reference terpenoids (α-, β-pinenes, sabinene, myrcene, *p*-cymene, α-terpinene, thymol, neryl + geranyl acetate, β-caryophyllene, α-humulene, caryophyllene oxide) and C_8_-C_28_ n-alkane series; and mass spectra with corresponding data in the literature [57] and computer mass spectra libraries (Wiley and NBS 54K). Identification has been approved when computer matching with the mass spectral libraries was with probabilities above 90%. 

### 4.6. Toxicity Test 

The toxicity of eight samples of *Rh. tomentosum* inflorescence and shoot essential oils was tested in vivo, using brine shrimp *Artemia* sp. (larvae) [74]. The eggs of shrimps hatch in sea water (31 g/L sea salt) at 20–25 °C within 48 h to provide larvae (nauplii). Thereafter, different concentrations of marsh rosemary essential oils dissolved in dimethyl sulfoxide (DMSO) were added, and survivors were counted after 24 h. Lethality (LC_50_ and LC_95_) of nauplii was calculated (*n* ≥ 4, with 95% confidence interval). Control tests were performed both with salt water and salt water with DMSO (5–40 µL). 

### 4.7. Antioxidant Activity

#### 4.7.1. ABTS^•^^+^ Assay

The stock solution containing ABTS^•^^+^ (2,2′-azino-bis(ethylbenzotiazoline-6-sulfonic acid) diammonium salt) and potassium persulfate (K_2_S_2_O_8_) was prepared, dissolving these materials in a mixture of methanol and water (80:20) and left for 12 h in the darkness. The working solution was prepared by diluting stock solution with a mixture of methanol and water (80:20) to obtain an absorbance value of 0.730 ± 0.02 at 734 nm. The absorbance was measured using the spectrophotometer (UV/Vis Lambda 25, Perkin Elmer Inc., Hamburg, Germany). Essential oils for analysis were diluted 1:50 with a mixture of methanol and water (80:20); 0.1 mL of prepared sample was allowed to react with 3.9 mL of working ABTS^•^^+^ solution for 15 min in the dark. Thereafter, the absorbance of reacted mixture was measured. The results are expressed in mmol/L TROLOX equivalents. All measurements were done in triplicate.

#### 4.7.2. DPPH^•^ Assay

A 6 × 10^−5^ M stock solution of DPPH^•^ was obtained by dissolving 2,2-diphenyl-1-picrylhydrazyl with methanol. The working solution was prepared by diluting stock solution with methanol to obtain an absorbance value of 0.730 ± 0.02 at 515 nm. Essential oils for analysis were diluted 1:200 with a mixture of methanol and water (80:20); 0.1 mL of the prepared sample was allowed to react with 3.9 mL of working DPPH^•^ solution for 30 min in the dark. Thereafter, the absorbance of reacted mixture was measured at 515 nm. Results are expressed in mmol/L TROLOX equivalents. All measurements were done in triplicate.

#### 4.7.3. TROLOX Equivalent Assay

First, 5 mg of Trolox ((±)-6-hydroxy-2,5,7,8-tetra-methylchromane-2-carboxylic acid) was dissolved in a mixture of methanol and water (70:30) and diluted to 100 mL. To obtain standard calibration curves, the solutions of four concentrations (200, 100, 50, and 25 mmol/L) of Trolox were prepared. Then, 0.1 mL of each Trolox solution was allowed to react with 3.9 mL of working solutions of ABTS^•^^+^ and DPPH^•^. Absorbance values were measured after 15 and 30 min at 734 and 515 nm, respectively. Linear calibration curves (see Figure S2) were obtained and their parameters were used for further calculations of antioxidant activity. All measurements were done in triplicate.

### 4.8. Antifungal Activity

#### 4.8.1. Agar Disc Diffusion Method for Testing Antifungal Activity of EO against Yeast

Pathogenic yeast *Candida parapsilosis* CBS 883C was from the collection of the Laboratory of Biodeterioration Research, Nature Research Centre (Vilnius, Lithuania). Baker’s yeast *S. cerevisiae* was obtained from a local market (shelf life not less than 2 weeks as specified by the producer). The antifungal activity of *Rh. tomentosum* was tested by the agar disc diffusion assay. The tests were carried out employing 100 μL of suspension containing 10^6^ CFU (colony-forming units)/mL of yeast spread on Mueller–Hinton agar (Liofilchem, Roseto, Italy) on a Petri plate (90 mm). Inoculum was obtained from yeasts cultured in Sabouraud dextrose agar (Liofilchem, Roseto, Italy) at 28 ± 1 °C for 48 h. After the absorption of inoculum by agar, the sterile paper discs (6 mm in diameter) impregnated with 10 µL of methanolic solution (10 mg/mL) of *Rh. tomentosum* EO (8 Sh, October) was placed on the surface of agar layer. The inoculated Petri plates were incubated at 30 °C for 48 h. Nystatin (100 I.U., Liofilchem, Roseto, Italy) was used as a positive control. The diameters of transparent inhibition zones formed in the yeast lawns around impregnated paper discs were used as a measure of antifungal activity. Each assay was repeated twice.

#### 4.8.2. Amperometric Study of *Rh. tomentosum* Essential Oil Effect on *S. cerevisiae* Membrane Permeability 

L-lactic was purchased from Riedel de Haen. Potassium ferricyanide was obtained from Fluka. Phosphate buffer was prepared from 0.1 mol/L KH_2_PO_4_ and contained 0.1 mol/L KCl (both from Fluka). The value of pH was adjusted with KOH.

Plain carbon paste was prepared by mixing 100 mg of graphite powder (Merck) with 50 µL of paraffin oil (Fluka). The paste was packed into an electrode body consisting of a plastic tube (diameter 2.9 mm) and a copper wire serving as an electrode contact. Yeast-modified electrodes were prepared by placing 10 µL of yeast suspension containing 40 mg of yeast and 0.5 mL water. The electrodes were allowed to dry at room temperature. The yeast-modified electrodes were transferred into custom-made camera (30 mL) containing a small amount of *Rh. tomentosum* EO (8 Sh, October) and subjected to the vapors of the oil for 5, 10, 20, 30 or 40 min. Thereafter, the electrodes were covered with a dialysis membrane (Aldrich-Sigma) presoaked in water. Yeast-modified electrodes without vapor pretreatment were used for comparison. The experiments were performed in triplicate.

Electrochemical experiments were carried out with a BAS-Epsilon Bioanalytical system (West Lafayette, IN, USA), and a three-electrode cell arranged with a magnetic stirrer. A modified carbon paste electrode served as a working electrode. Platinum wire and Ag/AgCl, 3N NaCl were, respectively, counter- and reference electrodes. Amperometric measurements were performed in a stirred phosphate buffer at pH 7.3 containing 0.5 mmol/L of K_3_[Fe(CN)_6_]. The yeast-modified electrode was held at a constant potential until a steady state of the background current was achieved. Electrode responses to lactic acid were measured after the addition of L-lactic acid to a final concentration of 0.2 mmol/L. All experiments were carried out at room temperature.

### 4.9. Statistical Analysis

The collected data were subjected to a one-way analysis of variance (ANOVA); the results were expressed as mean values, range intervals, and standard deviation (SD) values.

## 5. Conclusions

Depending on the plant growth stages, essential oils obtained from *Rh. tomentosum* plants growing wild in Lithuania varied quantitatively and qualitatively. According to the major compounds, the investigated oils could be attributed to the palustrol + ledol or palustrol + ledol + ascaridol chemotypes.

The differences in the biological activities of essential oils were ascribed to variation of oils compositions. Toxicity test in vivo using a brine shrimp (*Artemia* sp.) bioassay revealed the notable toxic activity of all investigated *Rh. tomentosum* essential oils containing ledol and palustrol as two major compounds. The inflorescence oil comprising a significant amount of myrcene and minor amounts of ascaridol was the least toxic among the investigated samples. Essential oils obtained from young shoots collected in May (third major compound ascaridol and a minor amount of myrcene) were less toxic compared to oils obtained from aged shoots gathered in September and October. The latter oils differed in the quantities of the third major compounds (ascaridol and lepalol). Actually, not only the major constituents of the oils play the principal role in the toxic activity, the impact of less abundant constituents should be considered also. 

On the contrary, the highest abilities to scavenge ABTS^•^^+^ and DPPH^•^ radicals were found for the essential oils of young shoots. Inflorescence oil and oils obtained from aged shoots collected in autumn possessed lower radical scavenging activities. The results of both assays (ABTS^+^, DPPH^•^) correlated with each other.

An investigation of the antifungal activity of *Rh. tomentosum* EO against *C. parapsilosis* by the agar disc diffusion method revealed that EOs could be potential natural antifungal agents. Mediated amperometry at yeast *S. cerevisiae*-modified electrodes appeared to be fast and effective method to investigate the effect of vapors of *Rh. tomentosum* essential oil on yeast membrane permeability. Yeasts affected by oil vapors possessed three to four-fold higher membrane permeability compared to that of intact yeast cells.

Plant-based products contain a mixture of different compounds with different mechanisms of action that may work in synergism. Besides, the advantage of plant-based products is that they are available, eco-friendly, cheap, sustainable, possess fewer side effects, and have better biodegradability compared to chemical antimicrobial agents.

## Figures and Tables

**Table 1:** Main composition (%, including constituents with quantity above 3.0%) of essential oils of *Rh. tomentosum* (Samanis forest marshes, Utena district, Eastern Lithuania).

Compounds	RI _DB-5_^#^ _Exp/Lit_	RI _HP-FFAP_^##^ _Exp/Lit_	1 Sh April	SD (*n* = 5)	2 Sh May	SD (*n* = 4)	3 Fl May	SD (*n* = 3)	4 Sh June	SD (*n* = 4)	5 Sh July	SD (*n* = 5)	6 Sh August	SD (*n* = 4)	7 Sh September	SD (*n* = 5)	8 Sh October	SD (*n* = 4)
Myrcene	991/990	1160/1160	1.8 ± 0.2	0.15	0.8 ± 0.1	0.05	**10.1** ± 1.3	1.30	**7.2** ± 0.3	0.25	1.8 ± 0.2	0.18	0.4 ± 0.1	0.10	1.8 ± 0.1	0.09	0.5 ± 0.1	0.08
*p*-Cymene	1025/1024	1270/1272	2.7 ± 0.2	0.28	2.8 ± 0.1	0.06	1.5 ± 0.2	0.28	1.8 ± 0.4	0.35	4.0 ± 0.4	0.29	4.8 ± 0.2	0.16	2.1 ± 0.1	0.08	0.2 ± 0.2	0.17
2-Methyl-6-methylene-3,7-octadien-2-ol	1095/1095	1630/1630	1.3 ± 0.4	0.49	0.5 ± 0.2	0.17	2.2 ± 0.2	0.21	2.6 ± 0.3	0.25	1.5 ± 0.2	0.15	2.8 ± 0.4	0.33	3.5 ± 0.6	0.45	2.6 ± 0.2	0.22
2-Methyl-6-methylene-1,7-octadien-3-ol	1158/1152	1700?	1.7 ± 0.3	0.24	1.5 ± 0.1	0.08	1.3 ± 0.1	0.10	0.1 ± 0.1	0.08	0.1 ± 0.1	0.08	1.8 ± 0.2	0.17	4.5 ± 0.4	0.30	1.7 ± 0.4	0.33
Lepalone ^a^	1258/1256	1757/1755	1.9 ± 0.2	0.22	2.7 ± 0.2	0.17	1.6 ± 0.4	0.40	2.3 ± 0.2	0.18	1.4 ± 0.2	0.17	2.4 ± 0.3	0.27	3.2 ± 0.2	0.16	3.5 ± 0.1	0.08
Lepalol ^b^	1282/1281	2035/2034	3.3 ± 0.3	0.26	2.3 ± 0.5	0.42	1.6 ± 1.1	1.10	0.7 ± 0.5	0.43	0.1 ± 0.1	0.09	0.1 ± 0.1	0.10	3.2 ± 0.6	0.46	**7.9** ± 0.3	0.28
Bornyl acetate	1290/1288	1578/1576	0.6 ± 0.1	0.10	0.7 ± 0.1	0.18	0.9 ± 0.1	0.12	3.3 ± 0.6	0.51	1.5 ± 0.6	0.55	2.8 ± 0.6	0.49	0.3 ± 0.1	0.08	-	
*iso*-Ascaridol *	1304/1307	1836/1836	3.0 ± 0.5	0.38	3.0 ± 0.2	0.16	1.4 ± 0.6	0.60	5.3 ± 1.6	1.31	**14.0** ± 2.4	1.83	**12.7** ± 2.0	1.65	**7.0** ± 2.4	1.97	0.1 ± 0.1	0.10
Palustrol	1569/1568	1920/1934	**33.5** ± 4.4	3.48	**31.3** ± 1.3	1.06	**30.0** ± 1.6	1.60	**24.6** ± 2.6	2.13	**25.2** ± 1.7	1.28	**26.2** ± 2.0	1.66	**26.0** ± 2.5	1.89	**30.4** ± 5.0	4.30
Ledol	1604/1602	2026/2043	**29.0** ± 5.0	4.06	**27.5** ± 2.0	1.64	**23.3** ± 2.3	2.30	**18.0** ± 2.9	2.47	**21.1** ± 2.5	2.06	**21.6** ± 3.0	2.45	**21.5** ± 4.0	2.86	**28.0** ± 2.1	1.90
Cyclocolorenone + *epi*-Cyclocolorenone	1770/1760	2326/2326	3.2 ± 2.0	1.61	4.1 ± 2.5	2.04	4.1 ± 1.6	1.33	4.0 ± 0.5	0.43	2.7 ± 0.5	0.36	3.0 ± 0.1	0.08	4.2 ± 0.5	0.40	6.2 ± 0.3	0.25
Total			95.2 ± 4.1	3.61	96.2 ± 2.6	0.35	91.0 ± 4.7	4.70	95.0 ± 2.0	1.66	95.2 ± 3.0	2.01	93.7 ± 4.3	3.69	96.2 ± 3.1	2.29	96.1 ± 2.3	1.95
Oxygenated sesquiterpenes			76.1 ± 4.5	3.18	67.7 ± 2.0	1.27	64.8 ± 2.3	2.30	56.8 ± 4.0	3.57	54.1 ± 1.5	1.11	59.6 ± 2.2	1.83	60.0 ± 3.0	2.12	75.9 ± 2.2	1.80

^a^ Lepalone = 2-Methyl-5(3-furyl)-pent-1-en-3-one. ^b^ Lepalol = 2-Methyl-5(3-furyl)-pent-1-en-3-ol. * Most probably it is *iso*-ascaridol + ascaridol (even alone ascaridol) as we evaluated in the previous work [13]. SD: standard deviation, *n* ≥ 3. List of minor (less than 3.0%) constituents: 4-methylene-5-hexenal, α-pinene, camphene, sabinene, β-pinene, (*E*,*E*)-2,4-heptadienal, α-terpinene, β-phellandrene, (*Z*)-β-ocimene, (*E*)-β-ocimene, γ-terpinene, terpinolene, *p*-cymenene, *p*-mentha-1,3,8-triene, 2-isopropyl-5-methyl-(2*Z*)-hexenal, lepaline, *allo*-ocimene, 2-methyl-6-methylene-1,7-octadien-3-one, *cis*-*p*-menth-2,8-dien-1-ol, *trans*-verbenol, ipsdienol, pinocarvone, terpinen-4-ol, *p*-cymen-8-ol, thuj-3-en-10-al, α-terpineol, myrtenal, γ-terpineol, 2-methyl-5-(3-furyl)-3-penten-2-ol, cumin aldehyde, thymol, citronellyl acetate, geranyl acetate, β-elemene, α-gurjunene, β-caryophyllene, β-copaene, β-gurjunene, γ-elemene, α-guaiene, aromadendrene, α-humulene, (*E*)-β-farnesene, *allo*-aromadendrene, *cis*-cadina-1(6),4-diene, 9-*epi*-β-caryophyllene, shyobunone, ledene, δ-cadinene, caryophyllene oxide, globulol, viridiflorol, *epi*-α-muurolol, α-cadinol, germacrone, *iso*-calamenediol. ^#^ Constituents are listed in order of their elution from non-polar DB-5 column; compounds are identified by their mass spectra and retention indices on both (polar HP-FFAP and non-polar DB-5) columns. RI_Exp_: Kovat’s indices determined experimentally on the non-polar column DB-5; RI_Lit_: Kovat’s indices for non-polar column DB-5 taken from literature [57]. ^##^ RI_Exp_: Retention indices determined experimentally on the polar column HP-FFAP; RI_Lit_: Retention indices for non-polar column are taken from webbook.nist.gov.

**Table 2:** Toxic activity of *Rh. tomentosum* essential oils.

*Artemia* sp. *nauplii* Lethality	1 Sh April (SD)	2 Sh May (SD)	3 Fl May (SD)	4 Sh June (SD)	5 Sh July (SD)	6 Sh August (SD)	7 Sh September (SD)	8 Sh October (SD)
LC_50_, µg/mL	14.06 (0.10)	16.10 (2.10)	20.50 (1.71)	13.59 (2.71)	14.43 (1.15)	15.97 (1.55)	11.23 (1.47)	11.73 (0.73)
LC_95_, µg/mL	55.95 (3.70)	46.94 (7.52)	76.07 (3.86)	34.66 (2.84)	41.58 (3.83)	43.90 (5.30)	34.00 (1.37)	34.83 (1.86)

SD: standard deviation, *n* ≥ 3.

**Table 3:** Antioxidant activity of *Rh. tomentosum* essential oils using ABTS^•^^+^ (2,2′-amino-bis(ethylbenzothiazoline-6-sulfonic acid) diammonium salt) assay.

EO	4 Sh June (SD)	5 Sh July (SD)	7 Sh September (SD)	8 Sh October (SD)
TROLOX (mmol/L)	48.19 ± 0.1 (0.01)	31.41 ± 0.2 (0.02)	30.35 ± 0.03 (0.03)	16.25 ± 0.2 (0.28)

SD: standard deviation, TROLOX: 6-hydroxy-2,5,7,8-tetra-methylchromane-2-carboxylic acid, *n* = 3.

**Table 4:** Antioxidant activity of *Rh. tomentosum* essential oils using DPPH^•^ (2,2-diphenyl-1-picrylhydrazyl) assay.

EO	2 Sh May (SD)	3 Fl May (SD)	4 Sh June (SD)	5 Sh July (SD)	6 Sh August (SD)	7 Sh September (SD)	8 Sh October (SD)
TROLOX (mmol/L)	11.92 ± 0.5 (0.5)	5.86 ± 0.07 (0.07)	19.89 ± 0.3 (0.03)	17.82 ± 0.5 (0.05)	12.07 ± 0.7 (0.7)	14.97 ± 0.5 (0.5)	12.26 ± 0.5 (0.5)

SD: standard deviation, *n* = 3.

## Supplementary Materials

The following are available online. Table S1. Summary of studies on bioactivities of *Rh. tomentosum* Harmaja (ex *Ledum palustre* L.) extracts, essential oils, or emitted volatiles. Figure S1. Geographical indication of *Rh. tomentosum* sampling site in Eastern Lithuania (Utena district). Figure S2. TROLOX standard calibration curves: (a) ABTS^•^^+^ assay; (b) DPPH^•^ assay.
