# Supplementary material for: Toxic, Radical Scavenging, and Antifungal Activity of Rhododendron tomentosum H. Essential Oils
Source: Molecules. 2020 Apr 5;25(7):1676. doi: 10.3390/molecules25071676 (PMC7181133; doi:10.3390/molecules25071676)
Supplement: Supplementary file 1 [file molecules-25-01676-s001.pdf]

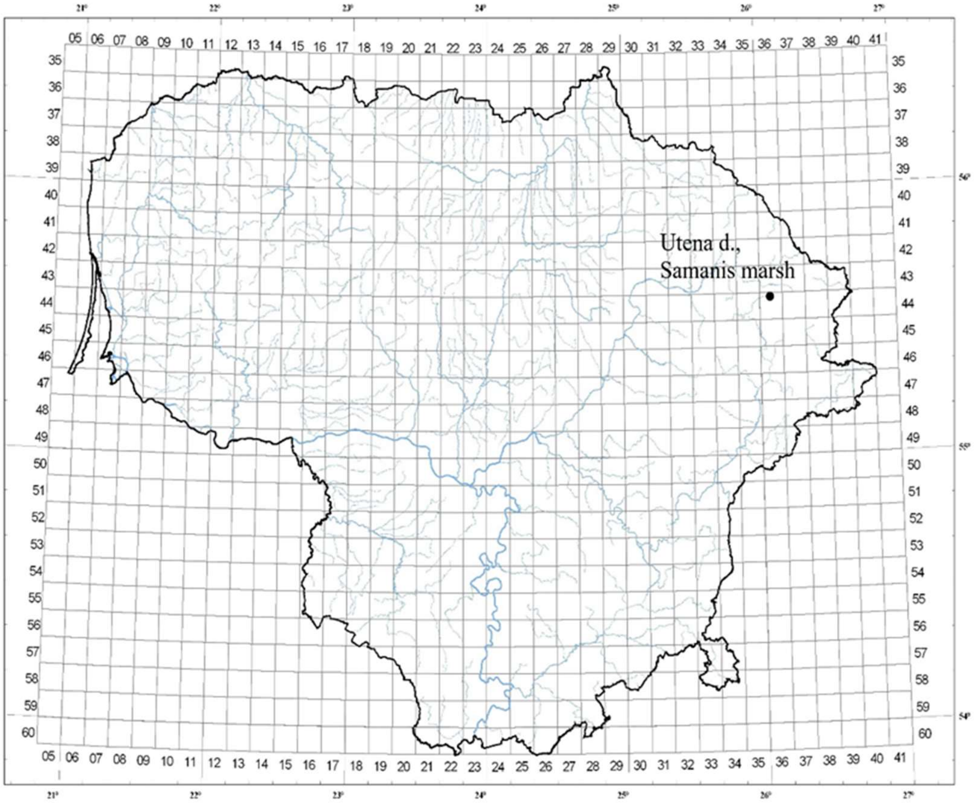

Figure S1. Geographical indication of *Rh. tomentosum* sampling site in Eastern Lithuania (Utena district).

Table S1. Summary of studies on bioactivities of *Rh. tomentosum* Harmaja (ex *Ledum palustre* L.) extracts, essential oils or emitted volatiles.

Activity, Evaluation Method	Product, Main Composition	Results
**Repellent** against host-seeking nymphs of *Ixodes ricinus* Linnaeus (Acari: Ixodidae) [1]	Extracts in solvents of different polarities and leaf EOs by steam distillation; myrcene, palustrol	EO diluted in acetone (10%) exhibited 95% repellency; extracts in ethyl acetate have shown >70% repellence activity. *I. ricinus* were deterred from feeding when 10% of EO was introduced.
**Repellent** against mosquitoes *Aedes aegypti* (L.) probing on treated cloth [2]	Extracts in solvents of different polarities, by steam distillation and SPME. Extract of leaf EO in ethyl acetate, *p*-cymene, sabinene, terpinyl acetate	Extracts significantly reduced activity of *Aedes aegypti* (L.): 83.5% repellency (0.5 ml plant extract).
**Insect growth regulating** and **toxic** effects on the metamorphosis stages of *Tenebrio molitor* (*Coleoptera, Tenebrionidae*), differential thermocouple calorimetry [3]	Leaves and flowers, extracts in 80% of EtOH (composition not indicated)	Non-lethal mild toxic effects of extracts. The timing of normal and failed ecdysis (after treatment with *L. palustre*), the length of intercdysial periods in *T. molitor* pupae, respiratory and muscular responses of poisoned insects was

		measured. The treated pharate pupae transformed into extra-pupal instars, which is a symptom of juvenilizing effect.
**Repellent** and **antifeedant** effects against *Hylobius abietis* L. and *Phyllodecta laticollis* Suffrian, olfactometry tests [4]	Fresh leaves, extracts in different organic solvents: ethyl acetate, MeOH and hexane; palustrol, ledol, β-myrcene	Feeding by the adults and larvae of *P. laticollis* and *H. abietis* was significantly reduced by methanol and hexane extracts.<br>Concomitant with less feeding, larval growth was retarded by ethyl acetate extract of the plant
**Repellence:** system *Brassica oleracea* var. *italica* (broccoli)–*Plutella xylostella* (crucifer specialist herbivore) – *Cotesia vestalis* (endoparasitoid of *P. xylostella*) is influenced by exposure to the natural semi-volatiles emitter plant *Rh. tomentosum* [5]	Semi-volatiles emitted from branches, main composition of emissions: palustrol (43%), β-myrcene (34%), aromadendrene (10%), ledol (9%)	*Rh. tomentosum*-exposed *B. oleracea* was less susceptible to *P. xylostella* oviposition at both night-time (12°C) and day-time (22°C) temperatures and less favoured and damaged by *P. xylostella* larvae at 12°C. Exposure did not interfere with indirect defence, *i.e.*, attraction of the natural enemy *C. vestalis* on host-damaged, *R. tomentosum*-exposed *B. oleracea* under 22°C, while there was a reduction in attraction (marginal preference towards host-damaged *B. oleracea*) under 12°C.
Biological **tests on growth** of *Lemna minor* L., and α-amylase activity in wheat seeds and coleoptile sections from 3-day-etiolated wheat sprouts [6]	Leaves and young shoots, EO adsorbed on natural zeolite	Volatile compounds completely inhibited the growth processes of the biotest used.
**Analgetic, Anti-inflammatory** [7]	EO, aqueous and MeOH extracts (flavonoid components)	Analgesic test: methanol extract (10.0 and 1.0mg/kg) and aqueous extract (10.0mg/kg) decreased the acetic acid-induced writhing response.<br>Anti-inflammatory test: MeOH extract (10.0 and 1.0mg/kg) and aqueous extract (10.0 mg/kg) decreased the paw edema in 2, 3, 4, 5 and 6 hours after lambda-carrageenan administration.
**Antibacterial** *in vitro,* broth dilution method, against *S. aureus, E. coli*, *P. aeruginosa*, *K. pneumoniae* [8]<br>**Anti-inflammatory** *in vivo*, carrageenan-induced edema in rats [8]	Shoots, aqueous extract and isolated polysaccharide complex<br>Shoots, dry aqueous extract, 40 and 70% in EtOH	Bacteriostatic effect at 1/160 dilution, excluding to *P. aeruginosa* (1/20 dilution) for polysaccharide complex.<br>51.5±4.6% Inhibition of edema by 40% EtOH extract, comparable to butadione.
**Anticancer** *in vitro*, mouse leukemia cells L1210 [9]	Aerial part, methylene chloride and MeOH extracts, Soxhlet	99% growth inhibition by methylene chloride extract, 71% growth inhibition by MeOH extract.

**Anticancer** *in vitro*, human lympho-blastoid Raji cells [10]	Aerial parts, crude EtOH (40%) extract	99% inhibition at 200μg/mL.
**Antidiabetic** *in vitro,* C2C12 murine skeletal myoblasts and the 3T3-L1 murine preadipocyte cell lines [11] **Antioxidant activity** *in vitro*, DPPH assay, ascorbic acid was used as the reference antioxidant [12]	Leaves, EtOH (80%) extract with 153.5 μg/mg total phenolic (chlorogenic acid, catechins, taxifolin, quercetin glycoside)	Cytoprotective properties under conditions of glucose toxicity (150mmol/L of glucose) and glucose deprivation (1.1mmol/L glucose) at 6.25μg/mL. Effect on the basal and insulin stimulated 3H-deoxy-glucose uptake in differentiated 3T3-L1 adipocytes at 50μg/mL. Triglyceride level was increased by 3-fold. Strong antioxidant activity, close to that of ascorbic acid.
**Antidiabetic** *in vitro,* Caco-2/15 cells; western blot analysis *in vivo*. Rats, oral glucose tolerance test [12]	Leaves, EtOH (80%) extract with 153.5μg/mg total phenolic	Instantaneous inhibition of differentiated Caco2/15 intestinal cells glucose absorption at 100μg/mL. Reduction of SGLT1 protein expression, AUC of blood glucose levels *in vivo*.
**Antifungal,** against *A. niger*, *C. albicans*, *M. canis*, *T. rubrum* and *T. mentagrophytes* [13]	EO	EO exhibited antifungal activities from mild to strong.
**Antifungal** *in vitro*, micro-broth dilution method, against *C. neoformans*, *S. cerevisiae*, *A. niger*, *C. albicans* [14]	Leaves, quercetin 3-β-D-(6-*p*-coumaroyl) galactoside and quercetin 3-β-D-(6-*p*-hydroxy-benzoyl)	MIC 16-63μg/mL for *C. neoformans, S. cerevisiae* and *A. niger*. MIC 250μg/mL for *C. albicans*. MIC 0.16-10μg/mL for amphotericin B and fluconazole.
**Anti-inflammatory,** subcutaneous carrageenan injection-induced hind paw oedema in rats [15].	Aerial parts, EO by supercritical fluid extraction (SFE) and hydrodistillation (HD). Palustrol (41.0-43.4%), ledol (23.3-26.7%), ascaridole (4.5-15.1%)	EO enhanced a significant inhibition of oedema (50-73%) for HD oil and (52-80%) for SFE oil. These results were similar to those obtained with piroxicam (70%) and ketoprofen (55%).
**Anti-inflammatory** *in vitro,* prostaglandin-synthesizing cyclooxygenase system from sheep seminal vesicles [16]	Aerial part, EO	EO inhibited cyclooxygenase *in vitro* 46.6% and carvacrol was responsible for 94% inhibition. Ledol did not inhibit the enzyme.
**Anti-inflammatory** *in vitro,* prostaglandin biosynthesis assay; PAF-induced exocytosis [17]	Aerial parts, aqueous extract, lyophilized	Moderate inhibition of prostaglandin biosynthesis (50%) and platelet activating factor (PAF)-induced exocytosis (71%).
**Antimicrobia**l *in vitro,* against *S. aureus*, S. *pneumoniae, C. perfringens*, *B. cereus*, *E. aerogenes*, *K. pneumoniae*, *C. albicans*, *M. smegmatis*, *A. lwoffii* and .*C krusei* [18]	EO and MeOH extracts, EO main composition: sabinene (17.8%), terpinen-4-ol (7.6%) and myrtenal (7.4%)	EO possessed antioxidant and low antimicrobial properties *in vitro*, while the water-insoluble parts of the methanolic extracts exhibited slight or no antimicrobial activity. EO strongly reduced DPPH* (IC_{50}=1.56μg/ml) formation and exhibited a hydroxyl radical scavenging

**Antioxidant** *in vitro*, DPPH assay, Fe^{3+}-EDTA-H_2O_2 deoxyribose assay, nonenzymatic lipid peroxidation of rat liver homogenate [18]		effect in the deoxyribose system (IC_{50}= 2.7μg/ml), and inhibited the nonenzymatic lipid peroxidation of rat liver homogenate (IC_{50}= 13.5μg/ml). The polar phase of the extract showed antioxidant activity.
**Antimicrobial** *in vitro*, against *Vibrio parahaemolyticus* [19]	Aerial parts, EO: α-thujenal (22.5%), β-phellandrene (10.3%), benzene,1-methyl-3-(1-methylethyl) (6.6%)	EO at concentration 5g/L inhibited growth of *V. parahaemolyticus* obviously.
**Anti-proliferative** and **pro-apoptotic** activity *in vitro*, the influence of EOs on blood lymphocytes'proliferation and apoptosis rates of synovia-derived cells was determined by flow cytometry [20]	Shoot EOs of γ-terpineol and palustrol/ledol chemotypes and microshoots cultivated *in vitro* of ledene oxide type	EOs had anti-proliferative and pro-apoptotic activity toward CD4 and CD8 T cells, synovia-infiltrating monocyte/macrophages and fibroblast-like synovial cells.<br>At 1:400 dilutions, all tested EOs increased the number of necrotic cells in synovial fibroblasts from RA synovia; and increased proportions of late apoptotic cells in leucocyte populations.
**Antithrombin** *in vitro*, thrombin solution from bovine plasma [21]	Aerial parts, MeOH extract, Soxhlet	88% of thrombin inhibition
**Hepato-protective** *in vivo*, rats, mice, CCl4 intoxication [22]	Shoots, dry extract (EtOH 40%)	Extract reduced hexobarbital sleeping time 1.4-and 3.2-fold, respectively, for rats and mice; and improved functional-metabolic and morphological parameters of liver.
**Toxicity** *in vivo*, tested on mice [23]	Shoots, 40% EtOH extract, chloroform and hexane fractions of EtOH extract	LD_{50}=2.800–3.200mg/kg for 40% EtOH extract after intraperitoneal administration, and no mortality of mice was after intragastric administration of extract at the dose of 10g/kg.<br>LD_{50} for the chloroform fraction 350mg/kg (intraperitoneal) and 2.600mg/kg (intragastric), LD_{50}=420mg/kg (intraperitoneal) and LD_{50}=5.100mg/kg (intragastric) for hexane fraction.
**Radioprotective** *in vivo*, mice irradiated with γ-irradiation [24]	Aerial parts, combination with *Archangelica officinalis* extracts	100% of animals survived after a dose of 6Gy (LD50/30); 70% survived after a dose of 7.5Gy (LD90/30), and 25% after a dose of 8Gy (LD100/12) by 30 day.
**Radioprotective** *in vivo*, 30 days albino mongrel male mice irradiated with γ-irradiation ($LD_{90/30}$) [25]	Aerial parts, combination with *Archangelica officinalis* extracts	Number of mouse pups was 10.2±0.6 in experimental and 7.4±0.7 in non-irradiated groups. The number of both sexes in the posterity of non-irradiated parents was equal; the number of female pups was 2.3 times larger than that of males.

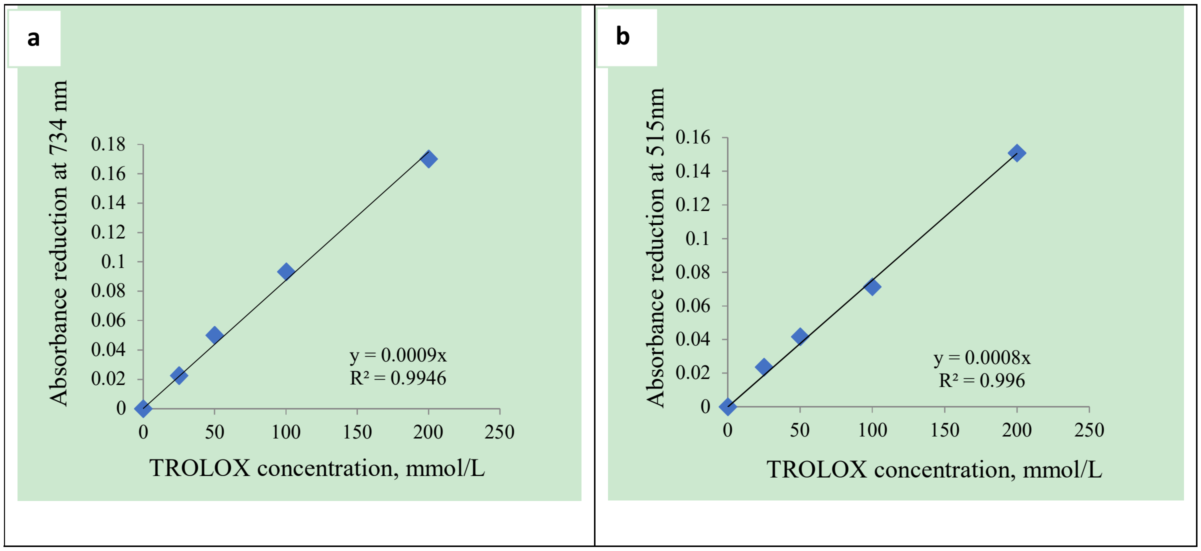

Figure S2. TROLOX standard calibration curves: (**a**) ABTS$^{\bullet+}$ assay; (**b**) DPPH$^{\bullet}$ assay.

References

1. Jaenson, T.G.T.; Pålsson, K.; Borg-Karlson, A.-K. Evaluation of extracts and oils of tick-repellent plants from Sweden. *Med. Vet. Entomol.* **2005**, *19*, 345–352.
2. Jaenson, T.G.T.; Pålsson, K.; Borg-Karlson, A.-K. Evaluation of extracts and oils of mosquito (Diptera: *Culicidae*) repellent plants from Sweden and Guinea-Bissau. *J. Med. Entomol*. **2006**, *43*, 113–119.
3. Kuusik, A.; Harak, M.; Hiiesaar, K.; Metspalu, L.; Tartes, U. Studies on insect growth regulating (IGR) and toxic effects of *Ledum palustre* extracts on *Tenebrio molitor* pupae (*Coleoptera*, Tenebrionidae) using calorimetric recordings. *Termochim. Acta* **1995**, *251*, 247–253.
4. Egigu, M.C.; Ibrahim, M.A.; Yahya, A.; Holopainen, J.K. *Cordeauxia edulis* and *Rhododendron tomentosum* extracts disturb orientation and feeding behavior of *Hylobius abietis* and *Phyllodecta laticollis*. *Entomol. Exp. Appl.* **2011**, *138*, 162–174.
5. Himanen, S.J.; Bui, Th.N.T.; Mengistu, M.M.; Holopainen, J.K. Utilizing associational resistance for biocontrol: Impacted by temperature, supported by indirect defense. *BMC Ecol.* **2015**; *15*, 1–12.
6. Petrov, K.A. The effect of essential oils of *Ledum palustre* L., *Acorus calamus* L., *Artemisia jacutica* Drob. on the biotests growth. *Rast. Resursy* **1998**, *34*, 47–50. (In Russian)
7. Min, Z.; Jing-jing, Z.; Wen-bin, W.; Yun, Z.; Xing-lei, Z.; Wei-jing, Q. Analgesic and anti-inflammatory activities of extracts from *Ledum palustre* L. in mice. *Nat. Prod. Res. Dev*. **2010**, *22*, 326–329.
8. Belousov, M.V.; Saratikov, A.S.; Akhmedzhanov, R.R.; Berezovskaya, T.A.; Yusubov, M.S.; Dmitruk, S.E.; Basova, E.V. Pharmacological properties of *Ledum palustre* (*Ericaceae*) from Tomsk region. *Rast. Resursy* **2006**, *42*, 130–140. (In Russian)
9. Goun, E.A.; Petrichenko, V.M.; Solodnikov, S.U.; Suhinina, T.V.; Kline, M.A.; Cunningham, G.; Nguyen, C.; Miles, H. Anticancer and antithrombin activity of Russian plants. *J. Ethnopharm*. **2002**, *81*, 337–342.
10. Spiridonov, N.A.; Konovalov, D.A.; Arkhipov, V.V. Cytotoxicity of some Russian ethnomedicinal plants and plant compounds. *Phytother. Res.* **2005**, *19*, 428–432.
11. Harbilas, D.; Martineau, L.C.; Harris, C.S.; Adeyiwola-Spoor, D.C.; Saleem, A.; Lambert, J.; Caves, D.; Johns, T.; Prentki, M.; Cuerrier, A.; et al. Evaluation of the antidiabetic potential of selected medicinal plant extracts from the Canadian boreal forest used to treat symptoms of diabetes: Part II. *Can. J. Physiol. Pharm.* **2009**, *8*, 479–492.
12. Nistor Baldea, L.A.; Martineau, L.C.; Benhaddou-Andaloussi, A.; Arnason, J.T.; Lévy, É.; Haddad, P.S. Inhibition of intestinal glucose absorption by anti-diabetic medicinal plants derived from the James Bay Cree traditional pharmacopeia. *J. Ethnopharmacol.* **2010**, *132*, 473–482.
13. Belousova, N.I.; Dmitruk, S.E.; Khan, V.A. Antifungal properties of essential oils of *Ledum* L. *Khim. Farm. Zh.* **1989**, *23*, 317–319.
14. Jin, C.; Strembiski, W.; Kulchytska, Y.; Micetich, R.G.; Daneshtalab, M. Flavonoid glycosides from *Ledum palustre* subsp. *decumbens. Daru* **1999**, *7*, 5–8.

15. Baananou, S.; Bagdonaite, E.; Marongiu, B.; Piras, A.; Porcedda, S.; Falconieri, D.; Boughattas, N.A. Supercritical CO_2 extract and essential oil of aerial part of *Ledum palustre* L.–Chemical composition and anti-inflammatory activity. *Nat. Prod. Res*. **2015,** *29,* 999–1005.
16. Wagner, H.; Wierer, M.; Bauer, R. In vitro Hemmung der Prostaglandin-Biosynthese durch etherische Ole und phenolische Verbindungen. *Planta Med*. **1986**, *3*, 184–187.
17. Tunón, H.; Olavsdotter, C.; Bohlin, L. Evaluation of anti-inflammatory activity of some Swedish medicinal plants. Inhibition of prostaglandin biosynthesis and PAF-induced exocytosis. *J. Ethnopharmacol.* **1995**, *48*, 61–76.
18. Kim, D.; Nam, B. Extracts and essential oil of *Ledum palustre* L. and their antioxidant and antimicrobial activities. *J. Food Sci. Nutr.* **2006**, *11*, 100–104.
19. Zhang, L. Chemical composition and antibacterial activity of the essential oil from Chinese wild *Ledum palustre* L. on *Vibrio Parahaemolyticus. Int. J. Food Nutr. Sci.* **2017**, *4*, 8–12.
20. Jesionek, A.; Kokotkiewicz, A.; Mikosik-Roczynska, A.; Ciesielska-Figlon, K.; Luczkiewicz, P.; Bucinski, A.; Daca, A.; Witkowski; J.M.; Bryl, E.; Zabiegala, B.; et al. Chemical variability of *Rhododendron tomentosum* (*Ledum palustre*) essential oils and their pro-apoptotic effect on lymphocytes and rheumatoid arthritis synoviocytes. *Fitoterapia* **2019**, *139*, 104402.
21. Spiridonov, N.A.; Konovalov, D.A.; Arkhipov, V.V. Cytotoxicity of some Russian ethnomedicinal plants and plant compounds. *Phytother. Res.* **2005**, *19*, 428–432.
22. Belousov, M.V.; Saratikov, A.S.; Tshichalin, V.S.; Akhmedganov, R.R.; Berezovskaya, T.P.; Basova, E.V.; Yusubov, M.S. Hepatoprotective properties of *Ledum palustre* (Ericaceae) shoots dry extract. *Rast. Resursy* **2007**, *43*, 126–129.
23. Basova, E.V. Chemical and Pharmacological Study of Wild Rosemary. Ph.D. Thesis, Tomsk State University, Tomsk, Russia, 2004.
24. Narimanov, A.A. Reproductive capacity of male mice protected from supralethal effect of gamma-radiation by the administration of a mixture of extracts from *Archangelica officinalis* and *Ledum palustre*. *Radiobiologia* **1992**, *32*, 271–275.
25. Narimanov, A.A.; Myakisheva, S.N.; Kuznetsova, S.M. The radioprotective effect of *Archangelica officinalis* Hoffm. and *Ledum palustre* L. extracts on mice. *Radiobiologia* **1991**, *31*, 391–393.
